# Noninvasive rapid detection of metabolic adaptation in activated human T lymphocytes by hyperpolarized ^13^C magnetic resonance

**DOI:** 10.1038/s41598-019-57026-1

**Published:** 2020-01-13

**Authors:** Emine Can, Mor Mishkovsky, Hikari A. I. Yoshihara, Nicolas Kunz, Dominique-Laurent Couturier, Ulf Petrausch, Marie-Agnès Doucey, Arnaud Comment

**Affiliations:** 1 Laboratory of Functional and Metabolic Imaging, Ecole Polytechnique Fédérale de Lausanne, CH-1015 Lausanne, Switzerland; 2 Cancer Research UK Cambridge Institute, University of Cambridge, Li Ka Shin Center, Robinson Way, Cambridge, CB2 0RE United Kingdom; 3 OnkoZentrum, CH-8038 Zürich, Switzerland; 4 Department of Oncology, University Hospital Lausanne (CHUV) and University of Lausanne (UNIL), Lausanne, Switzerland; 5 General Electric Healthcare, Chalfont St Giles, Buckinghamshire, HP8 4SP United Kingdom

**Keywords:** Metabolic pathways, Translational research

## Abstract

The metabolic shift induced in human CD4^+^ T lymphocytes by stimulation is characterized by an upregulation of glycolysis, leading to an augmentation in lactate production. This adaptation has already been highlighted with various techniques and reported in several previous studies. We herein propose a method to rapidly and noninvasively detect the associated increase in flux from pyruvate to lactate catalyzed by lactate dehydrogenase using hyperpolarized ^13^C magnetic resonance, a technique which can be used for *in vivo* imaging. It was shown that the conversion of hyperpolarized ^13^C-pyruvate to ^13^C-lactate during the one-minute measurement increased by a mean factor of 3.6 in T cells stimulated for 5 days as compared to resting T cells. This method can be extended to other metabolic substrates and is therefore a powerful tool to noninvasively analyze T cell metabolism, possibly *in vivo*.

## Introduction

**Figure 1:**
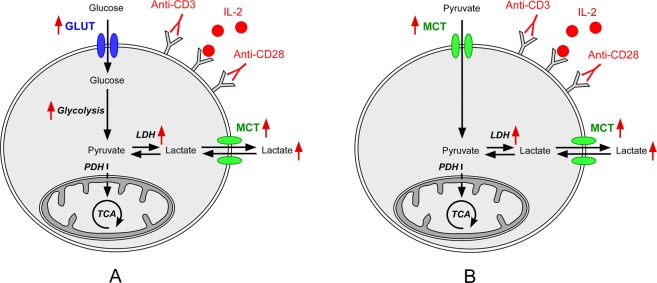
Main steps involved in the regulation of glucose (**A**) and pyruvate (**B**) metabolism in T cells. The red arrows highlight the metabolic adaptations.

Upon activation, T lymphocytes rapidly increase their requirements in metabolic substrates to sustain their proliferation and function^1^. While memory and naive T cells mostly rely on the β-oxidation of fatty acids to produce their ATP, it has been shown that the higher need in energy and biosynthetic material required by activated T cells leads to an upregulation of the catabolism of glucose^2^. This metabolic shift towards aerobic glycolysis leads to increased lactate production and is equivalent to the phenotype exhibited by cancer cells and first observed in 1956 by Otto Warburg^3^. This “Warburgian” behavior has been experimentally revealed in lymphocytes through several types of measurements: an increase in the expression of glucose and lactate transporters^4–9^, an increase in glucose uptake demonstrated with radioactive 2-deoxy-D-glucose^5,10^, an upregulation in enzymes involved in glycolysis, including lactate dehydrogenase (LDH)^11,12^, as well as the measurement of lactate accumulation either directly using enzymatic kits^5,13^, or indirectly by measuring the extracellular acidification rate (ECAR), which was shown to be highly correlated with the extracellular lactate production rate^14^. These metabolic adaptations are highlighted by the red arrows in Fig. 1A, which depicts the main steps involved in the regulation of glucose metabolism in T cells.

The most direct observation of variations in cellular glycolytic rate can be made through the measurement of the flux from glucose to pyruvate and lactate using isotopically labeled precursors. This is done either by determining the concentrations of ^14^C-pyruvate and ^14^C-lactate resulting from the uptake and metabolism of the radioactive precursor ^14^C-glucose^15^, or by ^13^C nuclear magnetic resonance (NMR), which provides a direct quantification of the metabolic products of ^13^C-glucose in intact cells^16^. The notable advantage of the latter technique is that the metabolites can be measured noninvasively and it can therefore be used *in vivo*, although the lack of sensitivity of ^13^C NMR severely limits its applications in humans. To partially circumvent this limitation, hyperpolarized (HP) ^13^C magnetic resonance (MR) technologies have been developed to increase the ^13^C signal intensity by several orders of magnitudes^17–19^. This enhancement originates from the large ^13^C nuclear spin polarization of the metabolic ^13^C-precursors, which is obtained using an instrument called a hyperpolarizer^20^. These preconditioned ^13^C-molecules are then delivered to cells, tissues, animals or humans, and the kinetics of formation of their downstream metabolic products can be measured in real-time using NMR or MR imaging (MRI)^21^. Although it has already been shown that ^13^C-glucose can be hyperpolarized and injected in rodents to detect glycolysis *in vivo*^22,23^, the current technology does not easily allow translation of this specific metabolic precursor from preclinical studies to human applications. Instead, ^13^C-pyruvate, a molecule that is actively taken up by cells using monocarboxylate transporters (MCTs) prior to being converted via LDH to ^13^C-lactate in the cytosol (Fig. 1B), has been shown to be an effective alternate HP ^13^C-probe to highlight the Warburg effect^19^. HP ^13^C-pyruvate has already been used to scan cancer patients at multiple sites in North America and Europe, and unlike 2-deoxy-2-(^18^F)fluoro-D-glucose (^18^F-FDG) positron emission tomography (PET), HP ^13^C MR allows glycolytic metabolism to be distinguished from oxidative phosphorylation, with the added advantage that it is non-radioactive^24,25^.

The aim of the present study is to demonstrate through *in vitro* measurements with human CD4^+^ T cells that HP ^13^C-pyruvate can detect the metabolic shift induced by the stimulation of T lymphocytes. Ensuring that this measurement modality is sufficiently sensitive to reveal the controlled activation of T lymphocytes in cell cultures is a necessary step towards *in vivo* experiments which avoids potentially confounding factors that could arise in the metabolically heterogeneous microenvironment of tissues.

## Materials and Methods

### Cell preparation

Healthy donor peripheral blood was obtained according to the declaration of Helsinki and upon written informed consent. This study was approved by the ethics committee of the Canton of Vaud, Switzerland, and the authors confirm that all experiments were performed in accordance with relevant guidelines and regulations. CD4^+^ T cells were isolated from peripheral mononuclear blood by immunomagnetic selection using a CD4 T cell negative depletion kit from Dynal (Dynal Biotech, Invitrogen). CD4^+^ T cell purity was >95%. T cells were isolated, grown and activated at the Ludwig Institute for Cancer research of Lausanne (Switzerland). Briefly, T cells were activated by exposure to anti-TCR/CD3 (OKT3, 10 µg/ml) antibodies coated on tissue culture plate and soluble anti-CD28 antibody (CD28.2, 1 µg/ml) in RPMI 1640 supplemented with 100 U/ml of IL-2 and 10% of fetal calf serum (FCS).

### Protocol for liquid chromatography–mass spectrometry

A set of activated as well as control resting T cells were incubated in RPMI 1640 supplemented with 100 U/ml of IL-2, 10% of FCS, and 5 mM sodium [2,3-^13^C_2_]pyruvate for 1 min at 37 °C. They were then snap frozen in liquid nitrogen to halt all metabolic activity. Diluted cell media (1:10) was extracted (20 µL) by the addition of cold MeOH:H2O (4:1, 80 µL)^26^. The extracts were centrifuged for 15 min at 14000 rpm at 4 °C and the resulting supernatant was transferred to liquid chromatography–mass spectrometry (LC-MS) vials for injection. Cell media extracts were analyzed by Hydrophilic Interaction Liquid Chromatography coupled to high resolution mass spectrometry (HILIC - HRMS) in negative ionization mode using a 6550 Quadrupole Time-of-Flight (Q-TOF) system interfaced with 1290 UHPLC system (Agilent Technologies) as previously described^27^.

### Protocol for mRNA sequencing

RNA was extracted and purified from cell lysates prepared from a second set of T cells in two different states (resting and activated) using an RNeasy Mini Kit (QIAGEN AG, Hombrechtikon, Switzerland). Pair-ended mRNA sequencing was performed on a HiSeq2500 (Illumina, San Diego, CA, USA). Purity-filtered reads were adapted and quality trimmed with Cutadapt^28^. Reads matching to ribosomal RNA sequences were removed with FastQ Screen (Babraham Institute, Cambridge, UK). Remaining reads were further filtered for low complexity with reaper^29^. Reads were aligned against Homo sapiens.GRCh38.92 genome using STAR^30^. The number of read counts per gene locus was summarized with htseq-count^31^ using Homo sapiens.GRCh38.92 gene annotation. Quality of the RNA-seq data alignment was assessed using RSeQC^32^.

### Sample preparation for MR measurements

A third set of activated as well as control resting T cells were transferred to the MR facility in an incomplete RPMI-1640 medium (modified with sodium bicarbonate, without methionine, cystine, or L-glutamine; Sigma-Aldrich, Switzerland) supplemented with 10% FCS in sterile plastic cell culture tubes containing 300 µL of medium. For each experiment, about 5·10^6^ of activated and their corresponding control resting T cells were incorporated into two separate 5-mm NMR tubes and unlabeled lactate solution was added to each tube to reach a final lactate concentration of 30 mM. The delay between the introduction of the cells into the NMR tubes in fresh medium and the completion of the hyperpolarized ^13^C MR measurements was less than 5 min. Cell viability tests indicated a survival rate of over 95% after 5 min in these conditions.

### Custom-designed MR probe

**Figure 2:**
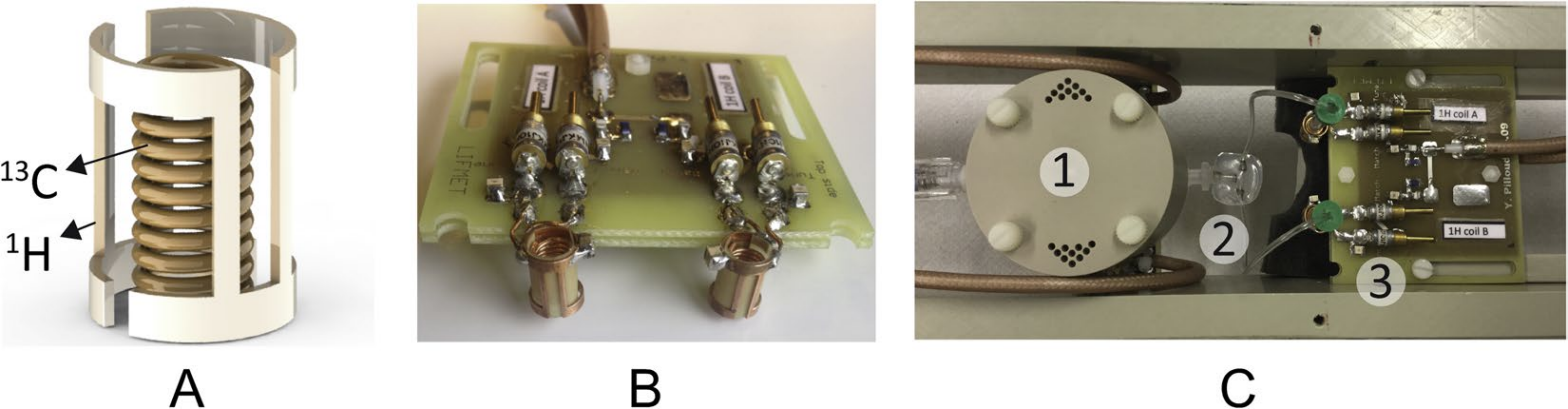
Custom-designed MR probe and injection pump. (**A**) Sketch of one of the two ^1^H/^13^C RF coil pairs adapted for 5-mm NMR tubes; (**B**) Picture of the MR probe highlighting the configuration of the two pairs of coils; (**C**) Top view of the injection pump (1) connected through a 2-way flow splitter (2) to two NMR tubes placed inside the MR probe (3).

The two NMR tubes containing the activated and resting T cells from a given donor were placed in a dual ^13^C MR probe custom-designed for a 9.4 T/31 cm horizontal bore magnet (Magnex Scientific, Abingdon, UK) (Fig. 2). The probe consists of two parallel single-turn saddle ^1^H radiofrequency (RF) coils each enclosing a multi-turn solenoid ^13^C RF coil designed for 5-mm NMR tubes (Fig. 2A). The two sets of coils are separated with an optimal distance (3 cm, center-to-center) that minimizes the inductive coupling while maintaining the coils close enough to the magnet isocenter (Fig. 2B). Each one of the two ^13^C coils is part of a resonant circuit, and both circuits are connected to the same coaxial cable through a PIN diode switch (Fig. S1A). The two ^1^H coils are also connected to a single coaxial cable through another PIN diode switch. With this configuration, the PIN diode switch allows one to automatically acquire data from the two tubes alternatively with a set delay (2 s in the present study) between each acquisition through a preprogrammed switching TTL signal (ON/OFF) incorporated within the pulse sequence (Fig. S1B).

The probe and NMR tubes were kept at 37 °C ambient temperature during the experiment using the MR-compatible fan module of small rodent heater system (SA Instruments, Inc. USA). Both NMR tubes were connected by a thin injection line to the output of a remotely-controlled custom-designed injection pump through a 2-way flow splitter (Fig. 2C)^33,34^. Prior to the HP ^13^C MR experiments, two NMR tubes filled with Dulbecco’s phosphate-buffered saline (PBS) 1X solution were inserted inside the RF coils and proton images were acquired to position the probe at the magnet isocenter. Static field inhomogeneities were individually corrected by manual shimming for each tube and the shim parameters were separately saved for both. The two NMR tubes filled with PBS 1X were then replaced by the NMR tubes containing the T cells and the probe was re-inserted into the magnet bore at the exact same location.

### Hyperpolarization

A volume of 3.5 µL frozen neat [1-^13^C]pyruvic acid (14.2 M; Sigma-Aldrich) doped with 20 mM trityl radical (Albeda, Denmark) was loaded together with 4.7 µL frozen 10 M NaOH solution in a custom-built dynamic nuclear polarization (DNP) hyperpolarizer operating at 7 T and 1.00 ± 0.05 K^35,36^. The [1-^13^C]pyruvic acid sample was polarized for 1.5 h using a 55-mW microwave irradiation set to 196.81 GHz. The solid-state ^13^C polarization build-up was monitored inside the hyperpolarizer by applying a 5° RF pulse every 5 min. Once polarized to 60 ± 5%, the frozen acid was rapidly dissolved in 6 ml preheated PBS to reach a physiologically compatible pH (~pH 7.5). The resulting hyperpolarized solution was automatically transferred into the injection pump.

### MR acquisition

After 300 μl of HP [1-^13^C]pyruvate solution was remotely injected within 3 s in each tube through the 2-way flow splitter, interleaved ^13^C MRS measurements were acquired on each sample alternatively (5° flip angle rectangular RF pulse with a repetition time of 4 s). The shim parameters optimized for each tube were automatically set in between each acquisition. All ^13^C MR measurements were performed with a Direct Drive spectrometer (Agilent, Palo Alto, CA, USA) interfaced to the actively-shielded 9.4 T / 31 cm horizontal bore magnet.

### Data analysis and statistics

Raw LC-MS files were processed in Profinder B.08.00 software (Agilent Technologies) using the targeted data mining in isotopologue extraction mode. The metabolite identification was based on accurate mass and retention time matching against an in-house database containing data on 600 polar metabolite standards (analyzed in the same analytical conditions). The Extracted Ion Chromatogram areas (EICs) of each isotopologue (M + , M + 1, M + 2 and M + 3) were corrected for natural isotope abundance^37^ and the label incorporation or ^13^C enrichment of lactate was calculated based on relative isotopologue abundance (in %), in each one of two analyzed conditions^38^.

The MR signal integrals were quantified from summed spectra using the peak fitting module from the OriginPro 2019 (OriginLab, USA) Peak Analyzer toolbox. The signal integral ratios between metabolic product ([1-^13^C]lactate) and substrate ([1-^13^C]pyruvate) were compared across all experiments with resting and activated T cells. The overlapping resonance from the impurity identified at 183 ppm was subtracted from the lactate peak using a protocol illustrated in Fig. S2.

Statistical analyses were performed on the data represented on a logarithmic scale. This was motivated by the fact that the variance of the lactate-to-pyruvate ratio increases with the mean, which is a typical characteristic of strictly positive data. From a biological point of view, we can also expect all resting T cells to exhibit a similar metabolic activity, whereas the stimulation may alter the T cells of each donor in a less homogenous manner. The paired (resting and activated condition for each donor) lactate-to-pyruvate ratios were analyzed using random-intercept linear mixed models with state (resting/activated) as fixed effect and patients as random effects. Linear mixed models have the Student’s paired T-test as special case (same estimates and inference) but comes with the following advantages: the model assessment can be performed on n = 8 (independent conditionally to the fixed and random effects) residuals instead of 4, and that the dependence between the paired observations can be quantified. The same statistical model was used to analyze the mRNA sequencing and LC-MS data.

## Results and Discussion

It has previously been shown that the mRNA expression of MCT1 and LDHA is strongly elevated in activated human T lymphocytes and that lactate concentration increases in the extracellular environment^39^. To verify that the cells used in the present study exhibited a similar behavior, mRNA sequencing and LC-MS analysis after incubation in a medium supplemented with [2,3-^13^C_2_]pyruvate were performed on sets of T cell cultures in two different states (resting and activated). Given that about 3% of the endogenous lactate bears one ^13^C atom while only 0.03% has two, doubly ^13^C-labeled pyruvate was used to ensure that its conversion to lactate could be measured by LC-MS with high sensitivity and accuracy.

**Figure 3:**
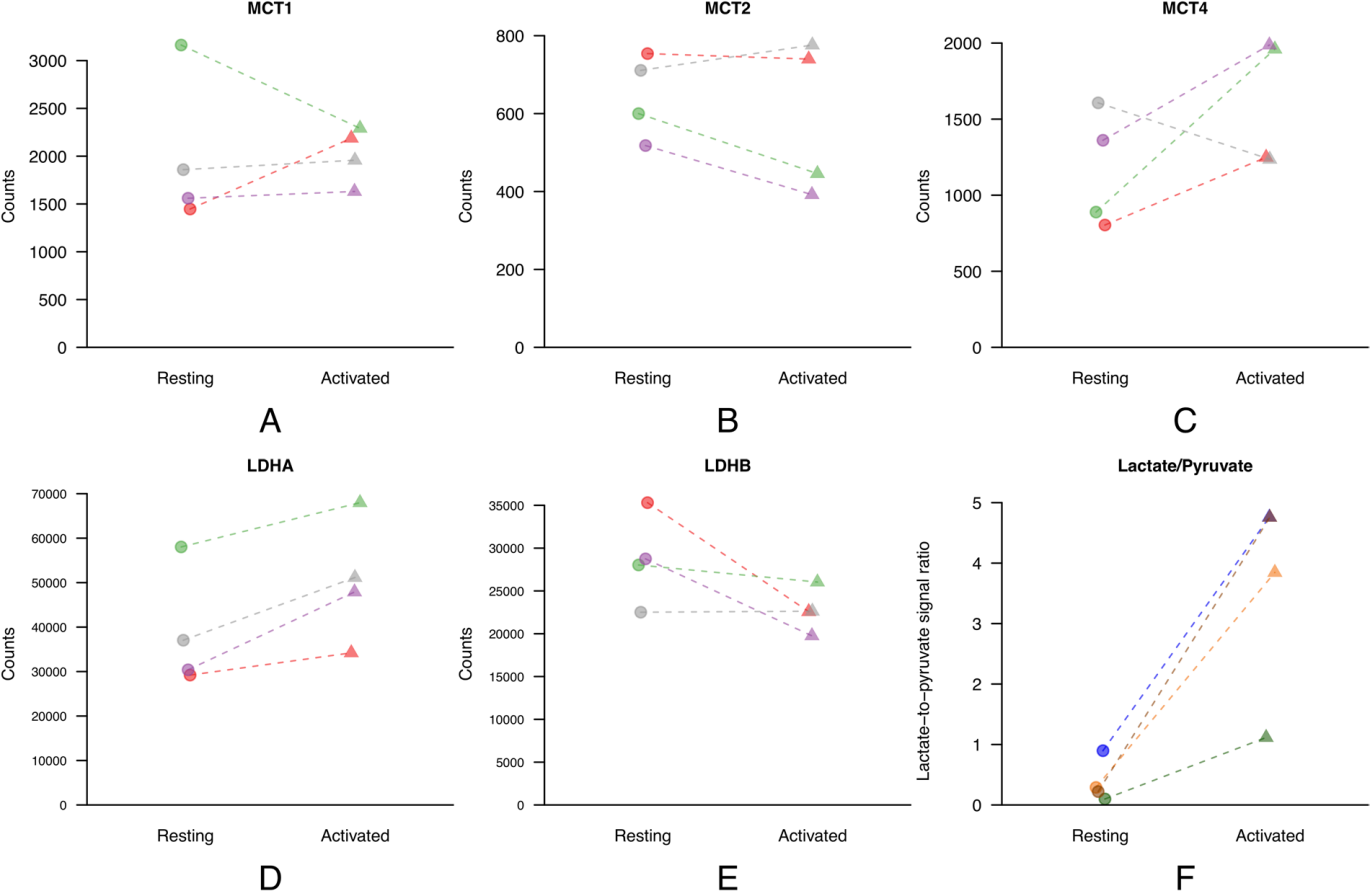
MCT1 (**A**), MCT2 (**B**), MCT4 (**C)**, LDHA (**D**), and LDHB (**E**) expression measured by mRNA sequencing on human T lymphocytes in both resting and activated states obtained from 4 donors. Each color corresponds to a different donor. Only the expression of LDHA was significantly different between the two states; **(F**) [2,3-^13^C_2_]lactate-to-[2,3-^13^C_2_]pyruvate ratio deduced from LC-MS experiments performed on human T lymphocytes from 4 other donors following the incubation of the cells in a medium containing 5 mM sodium [2,3-^13^C_2_]pyruvate for 1 min at 37 °C. The ratio significantly increases when comparing the activated to the resting state.

The mRNA analysis demonstrated that LDHA expression significantly increased upon activation (p = 0.032), while none of the MCTs were significantly more expressed in the activated state (Fig. 3A–E). This result is in good agreement with a previous study showing that LDHA stays highly overexpressed even after 6 days of stimulation while MCTs expression reduces from an early high expression within the first hours of stimulation to return to their resting level after such a long activation period^39^.

The statistical analysis of the LC-MS measurements shows a highly significant increase (p = 0.004) of the [2,3-^13^C_2_]lactate-to-[2,3-^13^C_2_]pyruvate ratio upon activation, with a 95% confidence interval of [4.54, 28.76] (Fig. 3F). For the 4 donors, the measured ratio was larger in activated T cells than in resting T cells. The mean enhancement factor was 11.4 and the paired observations were highly correlated (ρ = 0.74).

The higher LDHA expression together with the observed increase in [2,3-^13^C_2_]lactate-to-[2,3-^13^C_2_]pyruvate ratio can be associated with the upregulation of glycolysis triggered by the stimulation. Note that the residuals analysis of the linear mixed models for all the above-described measurements shows a good model fit (homoscedasticity and symmetry around 0; Fig. S3).

**Figure 4:**
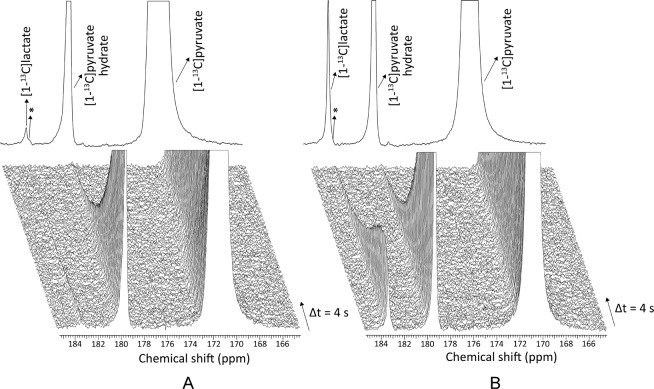
Representative time evolution of the ^13^C MR spectrum detected after the injection of HP [1-^13^C]pyruvate solution in resting (**A**) and activated (**B**) T cells. The delay between each acquisition was set to 4 s, starting 2 s after the beginning of the injection. The sum of the 1^st^ to 15^th^ spectra is displayed on the top. Along with the substrate resonance at 171.1 ppm, two products were detected: [1-^13^C]pyruvate hydrate at 179.5 ppm and [1-^13^C]lactate at 183.2 ppm. The impurity signal (marked with a star) overlapping with the lactate peak was subtracted from the spectra prior to quantification of the lactate signal.

**Figure 5:**
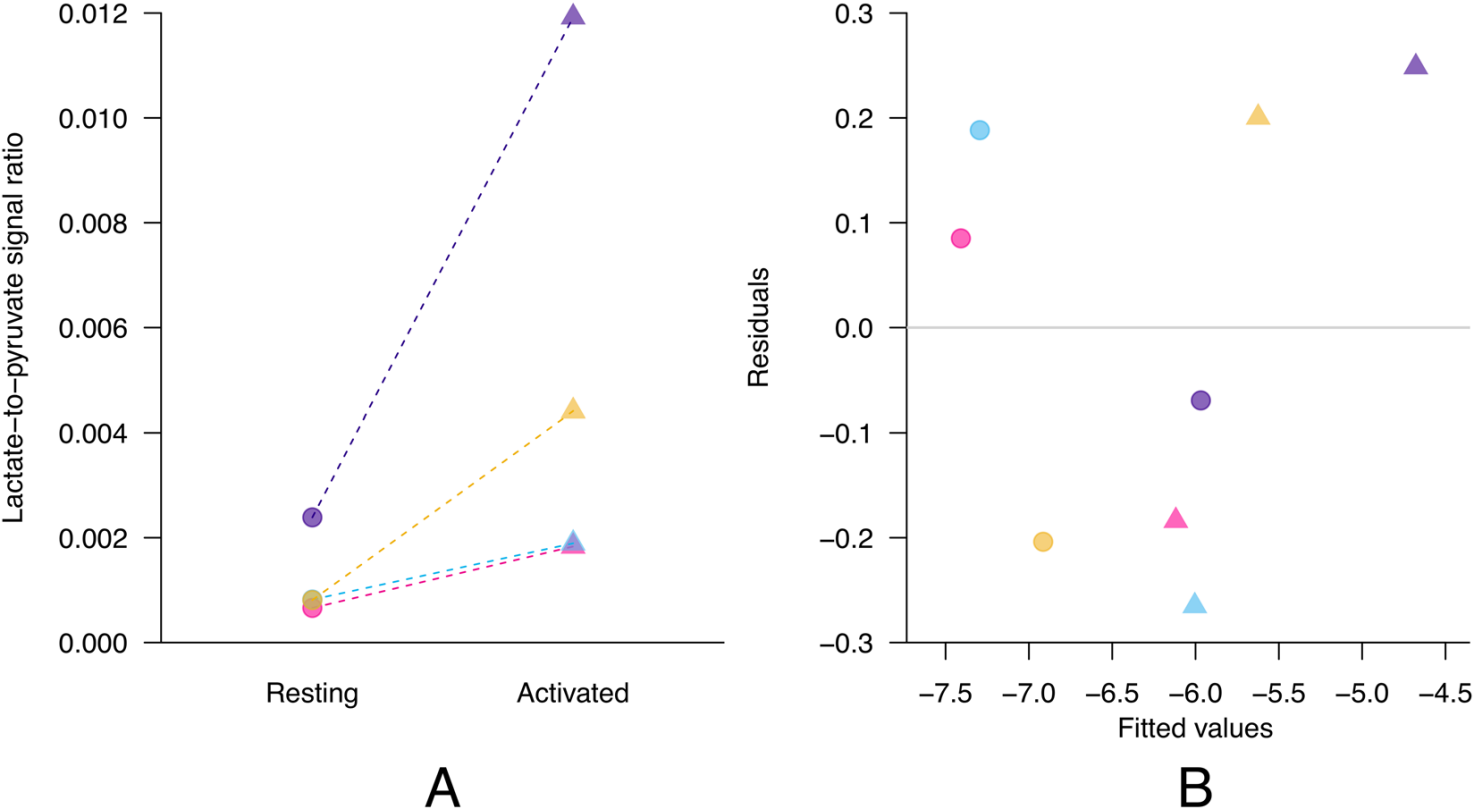
(**A**) Lactate-to-pyruvate ratio deduced from the hyperpolarized ^13^C MRS experiments performed on human T lymphocytes in two different states (resting and activated) obtained from 4 donors. Each color corresponds to a different donor. The lactate-to-pyruvate ratio significantly increases when comparing the activated to the resting state; (**B**) Residual analysis of the random-intercept linear mixed models fits of lactate-to-pyruvate ratio. The plot shows the residuals (y-axis) versus the fitted values (x-axis). Point colors correspond to donor and symbols to states: dots for resting and triangles for activated. These model checks, showing symmetry of the residuals around 0 and homoscedasticity, suggest a good fit of the model to the data.

In the MR measurements performed on another set of T cell cultures in two different states (resting and activated), the formation of [1-^13^C]lactate was readily detected following the injection of hyperpolarized [1-^13^C]pyruvate in both resting and *in vitro* activated T cells (Fig. 4). Although the observed lactate signal is the sum of both intra- and extracellular lactate, the line width of the resonance being too large to differentiate the two pools^40^, most of the detected signal likely originated from extracellular lactate. The [1-^13^C]pyruvate hydrate resonance was also detected. However, despite the relatively high SNR of the [1-^13^C]pyruvate resonance, no ^13^C-bicarbonate signal could be observed, which demonstrates that the flux through pyruvate dehydrogenase (PDH) was low and that only a minute fraction of the injected pyruvate was used to feed the tricarboxylic acid (TCA) cycle. In all 4 MR experiments, it was observed that the lactate-to-pyruvate signal ratio was larger in activated T cells than in resting T cells (Fig. 5A). This increase in ^13^C labeling of the lactate pool can be explained by an increased flux through LDH since the mRNA analysis showed that LDHA expression is upregulated in the activated state. It also correlates with the LC-MS data which demonstrated that, within a 1 min time window, the lactate labeled from pyruvate is significantly larger in the activated state. The statistical analysis of the MR data showed that the lactate-to-pyruvate signal ratio significantly increased (p = 0.009) with a mean enhancement factor of 3.6, with a 95% confidence interval of [1.85,7.15] and that the paired observations were highly correlated (ρ = 0.84). The residuals analysis of the linear mixed models fit of the lactate-to-pyruvate signal ratio on the logarithmic scale is presented in Fig. 5B, and as in the case of the mRNA sequencing and LC-MS data analysis, it exhibits a good model fit.

Thanks to the dual ^13^C MR probe used in this study, measurements in both activated and resting T cells from each donor could be done simultaneously, thereby ensuring that the two cell suspensions had been exposed to the same environmental conditions after having been transported to the MR facility. This is crucial since T lymphocytes are known to be sensitive cells that struggle to survive *ex vivo* for long periods of time, especially in depleted medium^41^. It can also be seen from Fig. 4 that the noise level in the sets of ^13^C spectra acquired from both sides of the dual probe is equivalent, which demonstrates that the sensitivity was identical in each pair of measurements done in parallel. Moreover, the mean [1-^13^C]pyruvate SNR of the first spectrum was very similar in all 8 measurements (2500 ± 350).

HP ^13^C MR is a well-adapted technique to study T cell metabolism since in optimized conditions the number of cells needed for a measurement in a standard NMR tube only need to be on the order of 10^6^, as in the present study, whereas previous ^13^C NMR studies required more than 10^9^ cells trapped inside agarose beads^16^. In addition, because long acquisition times are required to be able to observe the increased lactate production in activated T cell preparations using non-HP ^13^C MR (on the order of 30 min in ref. ^16^), it is necessary to carefully control the oxygen influx and possibly perfuse the cells to ensure that their state does not change during the acquisitions. By contrast, because the measurements only last for a few minutes, this is not necessary for HP ^13^C MR experiments.

The mean lactate-to-pyruvate ratio deduced from the HP ^13^C MR experiments is about 3 times smaller than the mean ratio deduced from the LC-MS measurements. This difference is most likely due to a convolution of several of the following factors: first and foremost, these two types of measurements were not performed on the same set of cells, and, as evident from the observed heterogeneity in the data, the ratio can dramatically vary across donors. Second, as previously highlighted by Witney *et al*.^42^, the measured conversion rate depends on both pyruvate and lactate extracellular concentrations and the exact initial concentrations may have slightly varied across experiments. Third, although the ^13^C MR data was integrated over a 1 min time window, the mixing within the NMR tubes was most likely not instantaneous following the injection of the HP ^13^C-pyruvate solution and the initial uptake and conversion rate was probably significantly slower than in the cells measured by LC-MS.

The choice of incubation time (5 days) was motivated by the fact that previous studies had shown that CD4^+^ T cells maintain an increased glycolytic activity during prolonged stimulation^39,43^. This does not mean that the lactate-to-pyruvate signal that can be observed using HP ^13^C-pyruvate is maximum after this particular activation period since, as shown in Fig. 1B, the lactate signal intensity will depend on the activities of both MCTs and LDH, and the expression of both these enzymes vary during this stimulation period. In fact, the data published by Renner *et al*. suggests that a 48 h stimulation period might lead to a higher lactate-to-pyruvate signal since the mRNA expression of both LDHA and MCT1 are higher after 48 h than after 6 day of stimulation^39^.

Since our experiments were not performed with glucose as substrate, it is not straightforward to compare our results with the data obtained in previous metabolic studies with T lymphocytes. Nevertheless, the four-fold increase in lactate production upon activation is in good agreement with numbers previously reported in studies performed with glucose, where a 3-7 fold increase was observed^39,44,45^. A more quantitative comparison between the techniques should be possible using HP ^13^C-glucose. The main conclusion of our study is that the metabolic adaptation triggered by T cell stimulation can be readily detected by HP ^13^C MR at much higher sensitivity than reported to date. The proposed method can complement the standard ECAR measurements with the advantage that metabolic fluxes are directly measured and that there is no need to add enzyme inhibitors. As illustrated in the present study, LC-MS can also be used to provide complementary measurements of metabolic fluxes using isotopically labeled substrates. However, the non-destructive nature of HP ^13^C MR makes it a unique tool for longitudinal *in vitro* studies.

Our dual parallel measurements (activated vs. resting T cells) could be extended to a larger number of parallel measurements using an integrated microfluidic system^46^, for instance to compare T cells that have been stimulated for a number of different time periods in a single measurement. HP ^13^C MR can also be used to directly and noninvasively analyze the behavior of T cells in presence of antigens, possibly *in vivo*. Since it is well known that cancer cells will also compete for pyruvate^21^, HP [1-^13^C]pyruvate might however not be the ideal substrate to detect T cell activation in the tumor microenvironment and alternative HP ^13^C tracers shall be tested. We therefore plan on extending the proposed method to other metabolic substrates such as fatty acid and glutamine, the uptake and metabolism of which is also known to be altered in T lymphocytes upon activation^11^.

## Supplementary information

Supplementary Information 

## Data Availability

All data is available from the authors upon reasonable request.

## References

[1] MacIver NJ, Michalek RD, Rathmell JC (2013). Metabolic regulation of T lymphocytes. Annu. Rev. Immunol..

[2] Pearce EL, Poffenberger MC, Chang C-H, Jones RG (2013). Fueling Immunity: Insights into Metabolism and Lymphocyte Function. Sci.

[3] Warburg O (1956). On the origin of cancer cells. Sci.

[4] Chakrabarti R, Jung CY, Lee TP, Liu H, Mookerjee BK (1994). Changes in glucose transport and transporter isoforms during the activation of human peripheral blood lymphocytes by phytohemagglutinin. J. Immunol..

[5] Frauwirth KA (2002). The CD28 signaling pathway regulates glucose metabolism. Immunity.

[6] Jacobs SR (2008). Glucose uptake is limiting in T cell activation and requires CD28-mediated Akt-dependent and independent pathways. J. Immunol..

[7] Macintyre AN (2014). The glucose transporter Glut1 is selectively essential for CD4 T cell activation and effector function. Cell Metab..

[8] Murray CM (2005). Monocarboxylate transporter MCT1 is a target for immunosuppression. Nat. Chem. Biol..

[9] Wang R, Green DR (2012). Metabolic reprogramming and metabolic dependency in T cells. Immunol. Rev..

[10] Ishimori T (2002). Increased (18)F-FDG uptake in a model of inflammation: concanavalin A-mediated lymphocyte activation. J. Nucl. Med..

[11] Ghesquière B, Wong BW, Kuchnio A, Carmeliet P (2014). Metabolism of stromal and immune cells in health and disease. Nat.

[12] Marjanovic S, Eriksson I, Nelson BD (1990). Expression of a new set of glycolytic isozymes in activated human peripheral lymphocytes. Biochim. Biophys. Acta.

[13] Marjanovic S, Wielburski A, Nelson BD (1988). Effect of phorbol myristate acetate and concanavalin A on the glycolytic enzymes of human peripheral lymphocytes. Biochim. Biophys. Acta.

[14] Jones N (2017). Metabolic Adaptation of Human CD4(+) and CD8(+) T-Cells to T-Cell Receptor-Mediated Stimulation. Front. Immunol..

[15] O’Rourke AM, Rider CC (1989). Glucose, glutamine and ketone body utilisation by resting and concanavalin A activated rat splenic lymphocytes. Biochim. Biophys. Acta.

[16] Bental M, Deutsch C (1993). Metabolic Changes in Activated T-Cells - an Nmr-Study of Human Peripheral-Blood Lymphocytes. Magn. Reson. Med..

[17] Ardenkjaer-Larsen JH (2015). Facing and Overcoming Sensitivity Challenges in Biomolecular NMR Spectroscopy. Angew. Chem. Int. Ed. Engl..

[18] Comment A (2016). Dissolution DNP for *in vivo* preclinical studies. J. Magn. Reson..

[19] Kurhanewicz J (2011). Analysis of Cancer Metabolism by Imaging Hyperpolarized Nuclei: Prospects for Translation to Clinical Research. Neoplasia.

[20] Balzan, R. *et al*. Dissolution Dynamic Nuclear Polarization Instrumentation for Real-time Enzymatic Reaction Rate Measurements by NMR. *J Vis Exp: JoVE*, 53548 (2016). 10.3791/53548 PMC4828185 26967906

[21] Comment A, Merritt ME (2014). Hyperpolarized Magnetic Resonance as a Sensitive Detector of Metabolic Function. Biochem.

[22] Mishkovsky M (2017). Measuring glucose cerebral metabolism in the healthy mouse using hyperpolarized 13C magnetic resonance. Sci. Rep..

[23] Rodrigues TB (2014). Magnetic resonance imaging of tumor glycolysis using hyperpolarized 13C-labeled glucose. Nat. Med..

[24] Kurhanewicz J (2019). Hyperpolarized C-13 MRI: Path to Clinical Translation in Oncology. Neoplasia.

[25] Marco-Rius, I. & Comment, A. In eMagRes (eds Harris, R. K. & Wasylishen, R. L.) (2019).

[26] Ivanisevic J (2013). Toward ‘omic scale metabolite profiling: a dual separation-mass spectrometry approach for coverage of lipid and central carbon metabolism. Anal. Chem..

[27] Gallart-Ayala H (2018). A global HILIC-MS approach to measure polar human cerebrospinal fluid metabolome: Exploring gender-associated variation in a cohort of elderly cognitively healthy subjects. Anal. Chim. Acta.

[28] Martin M (2011). Cutadapt removes adapter sequences from high-throughput sequencing reads. EMBnet.journal.

[29] Davis MPA, van Dongen S, Abreu-Goodger C, Bartonicek N, Enright AJ (2013). Kraken: A set of tools for quality control and analysis Of high-throughput sequence data. Methods.

[30] Dobin A (2013). STAR: ultrafast universal RNA-seq aligner. Bioinformatics.

[31] Anders S, Pyl PT, Huber W (2014). HTSeq—a Python framework to work with high-throughput sequencing data. Bioinformatics.

[32] Wang L, Wang S, Li W (2012). RSeQC: quality control of RNA-seq experiments. Bioinformatics.

[33] Cheng T (2013). Automated transfer and injection of hyperpolarized molecules with polarization measurement prior to *in vivo* NMR. NMR Biomed..

[34] Comment A (2007). Design and performance of a DNP prepolarizer coupled to a rodent MRI scanner. Concepts Magn. Reson..

[35] Cheng T, Capozzi A, Takado Y, Balzan R, Comment A (2013). Over 35% liquid-state 13C polarization obtained via dissolution dynamic nuclear polarization at 7 T and 1 K using ubiquitous nitroxyl radicals. Phys. Chem. Chem Phys.

[36] Yoshihara HA (2016). High-field dissolution dynamic nuclear polarization of [1-(13)C]pyruvic acid. Phys. Chem. Chem Phys.

[37] Midani FS, Wynn ML, Schnell S (2017). The importance of accurately correcting for the natural abundance of stable isotopes. Anal. Biochem..

[38] Roci I (2016). Metabolite Profiling and Stable Isotope Tracing in Sorted Subpopulations of Mammalian Cells. Anal. Chem..

[39] Renner K (2015). Metabolic plasticity of human T cells: Preserved cytokine production under glucose deprivation or mitochondrial restriction, but 2-deoxy-glucose affects effector functions. Eur. J. Immunol..

[40] Breukels V (2015). Direct dynamic measurement of intracellular and extracellular lactate in small-volume cell suspensions with C-13 hyperpolarised NMR. NMR Biomed..

[41] Traba, J., Miozzo, P., Akkaya, B., Pierce, S. K. & Akkaya, M. An Optimized Protocol to Analyze Glycolysis and Mitochondrial Respiration in Lymphocytes. J Vis Exp: JoVE (2016). 10.3791/54918 PMC5226256 27911401

[42] Witney TH, Kettunen MI, Brindle KM (2011). Kinetic modeling of hyperpolarized 13C label exchange between pyruvate and lactate in tumor cells. J. Biol. Chem..

[43] Akkaya B (2018). Increased Mitochondrial Biogenesis and Reactive Oxygen Species Production Accompany Prolonged CD4(+) T Cell Activation. J. Immunol..

[44] Gerriets VA (2015). Metabolic programming and PDHK1 control CD4(+) T cell subsets and inflammation. J. Clin. Invest..

[45] Hume DA, Radik JL, Ferber E, Weidemann MJ (1978). Aerobic Glycolysis and Lymphocyte-Transformation. Biochem. J..

[46] Jeong S (2017). Real-time quantitative analysis of metabolic flux in live cells using a hyperpolarized micromagnetic resonance spectrometer. Sci. Adv..

