# Supplementary material for: Noninvasive rapid detection of metabolic adaptation in activated human T lymphocytes by hyperpolarized 13C magnetic resonance
Source: Sci Rep. 2020 Jan 13;10:200. doi: 10.1038/s41598-019-57026-1 (PMC6957688; doi:10.1038/s41598-019-57026-1)
Supplement: Supplementary file 1 — Supplementary Information [file 41598_2019_57026_MOESM1_ESM.pdf]

Supplementary Material

Noninvasive rapid detection of metabolic adaptation in activated human T lymphocytes by hyperpolarized ^{13}C magnetic resonance

Emine Can[1], Mor Mishkovsky[1], Hikari A. I. Yoshihara[1], Nicolas Kunz[1], Dominique-Laurent Couturier[2], Ulf Petrausch[3], Marie-Agnès Doucey[4], Arnaud Comment[2,5,*]

[1] Laboratory of Functional and Metabolic Imaging, Ecole Polytechnique Fédérale de Lausanne, CH-1015 Lausanne, Switzerland

[2]Cancer Research UK Cambridge Institute, University of Cambridge, Li Ka Shin Center, Robinson Way, Cambridge CB2 0RE, United Kingdom

[3]OnkoZentrum, CH-8038 Zürich, Switzerland

[4]Department of Oncology, University Hospital Lausanne (CHUV) and University of Lausanne (UNIL), Lausanne, Switzerland

[5]General Electric Healthcare, Chalfont St Giles, Buckinghamshire HP8 4SP, United Kingdom

Corresponding Author

*E-mail: **arnaud.comment@ge.com**

ORCID

Arnaud Comment: 0000-0002-8484-3448

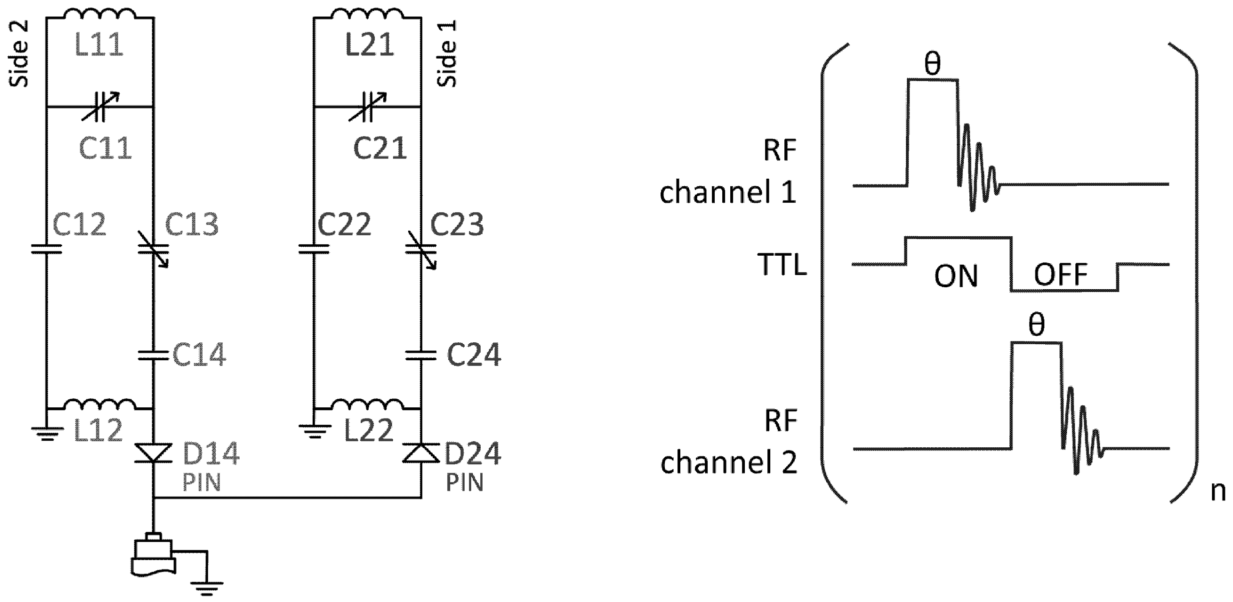

Figure S1. (A) Circuit schematic of one of the two symmetric channels (^{1}H and ^{13}C) of the custom-designed MR probe. The circuit scheme is identical for both ^{1}H and ^{13}C channels. The PIN diodes were placed in forward (D24) and reverse (D14) bias to isolate the two probe heads using a transistor-transistor logic (TTL) signal synchronized with the RF pulse via a digitally-controlled driver[1]. The capacitor values used in practice for C12 and C14 are 0.5 pF and 7.5 pF for ^{1}H and 6.2 pF and 102.2 pF for ^{13}C. The capacitor values for the other side of each channel are identical. L11 and L21 represents the inductors of the $^{1}H/^{13}C$ probe heads. Inductors (L12 and L22, 150nH each) were placed between the PIN diodes and the ground to create a DC current pass through; (B) A schematic diagram showing the pulse sequence implemented for alternating acquisitions of MR signals from each side of the probe (channel 1 corresponds to side 1 and channel 2 to side 2). A total of n acquisitions on each side is preset.

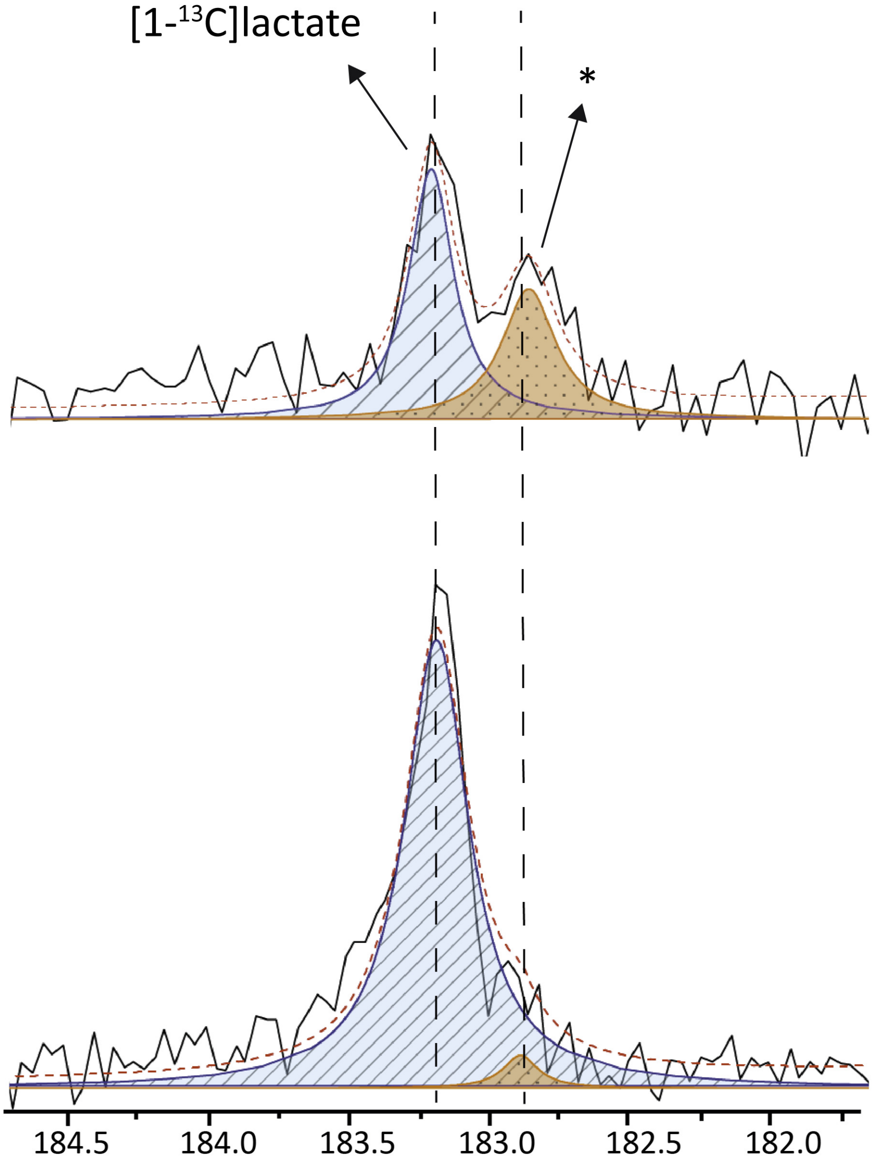

Figure S2. Representative spectra showing the impurity signal (marked with a star) overlapping with the lactate peak with a chemical shift difference of 0.3 ppm. To subtract the impurity signal from the [1-^{13}C]lactate signal, the peak fitting module from the OriginPro 2019 Peak Analyzer toolbox was used to fit both signals with a fixed chemical shift separation of 0.3 ppm for all experiments. Only the integral of the peak corresponding to the [1-^{13}C]lactate signal (light blue hatched area) was used for calculating the lactate-to-pyruvate signal ratios.

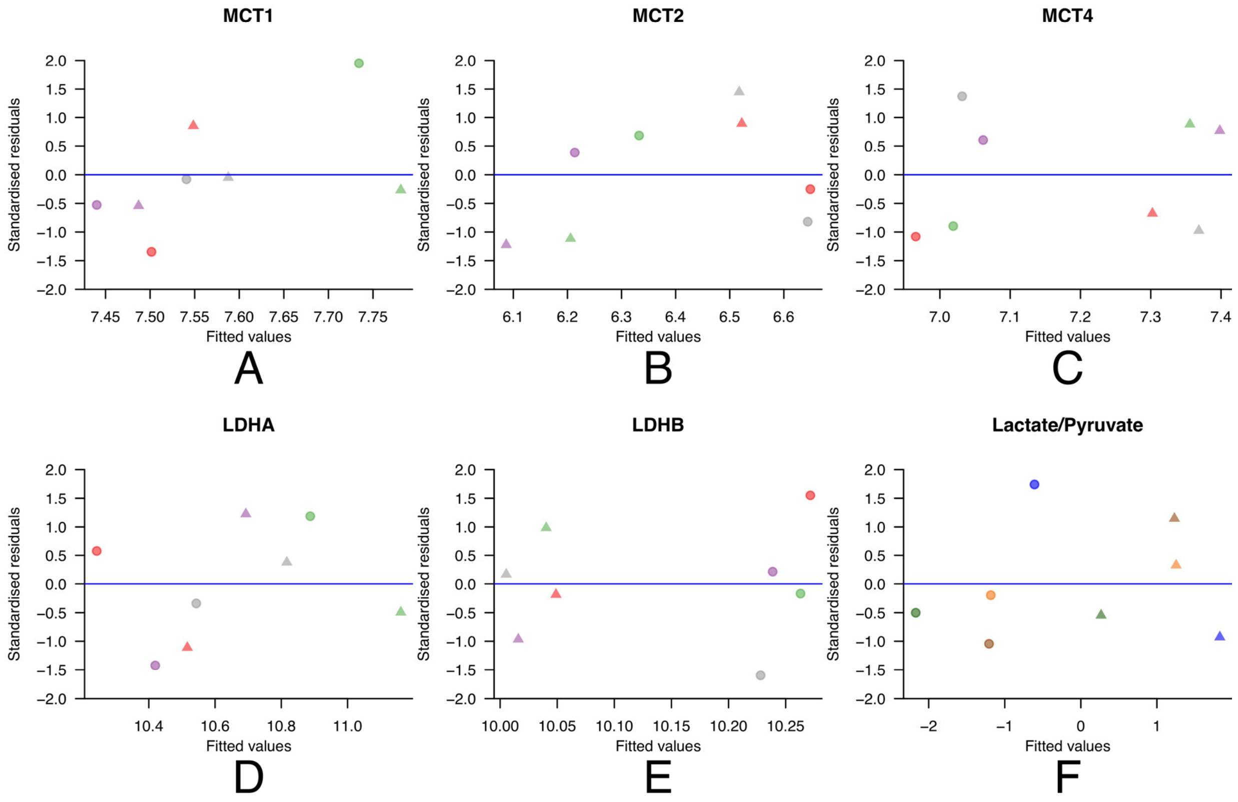

Figure S3. Residual analysis of the random-intercept linear mixed models fits of mRNA expression of MCTs (A-C), LDHA (D), LDHB (E), and [2,3-$^{13}C_2$]lactate-to-[2,3-$^{13}C_2$]pyruvate ratio measured by LC-MS. The plots show the residuals (y-axis) versus the fitted values (x-axis). Point colors correspond to donor and symbols to states: dots for resting and triangles for activated. These model checks, showing symmetry of the residuals around 0 and homoscedasticity, suggest a good fit of the model to the data.

References

1. Pilloud, Y. & Gruetter, R. in *ESMRMB 2012.* 556-557 (MAGMA).
